# Association of PI3K/AKT/mTOR pathway autophagy-related gene polymorphisms with pulmonary tuberculosis susceptibility in a Chinese population

**DOI:** 10.1590/0037-8682-0104-2023

**Published:** 2023-07-24

**Authors:** Juan He, Shengyuan Liu, Xujun Guo, Fan Zhang, Yuzheng Fan, Lijuan Wu, Howard Eugene Takiff, Yashuang Zhao

**Affiliations:** 1 Harbin Medical University, School of Public Health, Department of Epidemiology, Harbin, China. Harbin Medical University School of Public Health Department of Epidemiology Harbin China; 2 Shenzhen Nanshan Center for Chronic Disease Control, Department of Tuberculosis Control and Prevention, Shenzhen, China. Shenzhen Nanshan Center for Chronic Disease Control Department of Tuberculosis Control and Prevention Shenzhen China; 3 Laboratorio de Genética Molecular, CMBC, IVIC, Caracas, Venezuela. Laboratorio de Genética Molecular CMBC IVIC Caracas Venezuela

**Keywords:** PI3K/AKT/mTOR, Autophagy, Tuberculosis, SNP, Genetic risk score

## Abstract

**Background:**

Autophagy can inhibit the survival of intracellular microorganisms including *Mycobacterium tuberculosis* (*Mtb*)*,* and the PI3K/AKT/mTOR pathway plays a crucial role. This study investigated the association between PI3K/AKT/mTOR pathway autophagy-related gene polymorphisms and pulmonary tuberculosis (PTB) susceptibility.

**Methods:**

KEGG pathway and gene ontology (GO) databases were searched for genes belonging to the PI3K/AKT/mTOR and autophagy pathways. Thirty SNPs in nine genes were identified and tested for their associations with tuberculosis in 130 patients with PTB and 271 controls. We constructed genetic risk scores (GRSs) and divided the participants into 3 subgroups based on their GRSs:0-5, 6-10, and 11-16.

**Results:**

This analysis revealed that the *AKT1* (rs12432802), *RPTOR* (rs11654508, rs12602885, rs2090204, rs2589144, and rs2672897), and *TSC2* (rs2074969) polymorphisms were significantly associated with PTB risk. A decreasing trend was observed (*P* trend 0.020), in which a lower GRS was associated with a higher risk of PTB ([6-10] vs. [0-5]: OR (95%CI) 0.590 (0.374-0.931); [11-16] vs. [0-5]: OR (95%CI) 0.381 (0.160-0.906)).

**Conclusions:**

Polymorphisms in *AKT1, RPTOR,* and *TSC2* may influence susceptibility to PTB.

## INTRODUCTION

Pulmonary tuberculosis (PTB) is a communicable disease caused by *Mycobacterium tuberculosis* (*Mtb*). The latest World Health Organization (WHO) figures show that in 2021, there were approximately 10.6 million new TB patients globally^1^. *Mtb* is transmitted through the air, and close contacts of patients with active PTB have a high risk of being infected, although most patients will develop latent TB infections (LTBI). Individuals with LTBI have no clinical symptoms of TB, and nearly one-quarter of the global population harbors latent *Mtb* infections. About 5-10% of these individuals develop active TB at a later point in their lives when their immune system’s control of the latent infection fails^2^, making them a reservoir for new TB cases. For over thousands of years, *Mtb* has adapted to the human host and developed the ability to subvert host immune responses, survive within macrophages and cause diseases. Although the immune escape mechanisms of *Mtb* remain poorly understood, host genetic determinants have been shown to play a vital role in susceptibility to TB^3^. 

Autophagy, which inhibits the survival of intracellular microorganisms such as *Mtb*, is regulated by various signaling pathways^4^. A critical regulator of autophagy is the PI3K/AKT/mTOR pathway^5^. mTOR functions in two distinct multiprotein complexes: mTORC1 and mTORC2^6^. Activation of the PI3K/AKT pathway promotes *Mtb* survival by accelerating cell cycle progression and inhibiting apoptosis^7^. Activated AKT directly phosphorylates and stimulates mTORC1, blocking the formation of the unC-51-like autophagy kinase (ULK1) complex, thereby preventing the formation of autophagosomes and allowing *Mtb* to escape autophagic degradation in the host cell^7^. *Mtb* can also induce FoxP3+ Tregs to proliferate and be activated by inhibiting the PI3K/AKT/mTOR pathway as part of its immune escape repertoire^8^. Agents that inhibit the mTOR pathway, such as the anti-cancer agents Ibrutinib^9^ and Everolimus^10^, have been used as adjuvant host-directed therapeutics (HDT)^11^. Therefore, by studying this pathway, novel therapeutic targets may be identified.

Genomic techniques^12^ can identify single nucleotide polymorphisms (SNPs) that are markers for genes associated with complex diseases. Although polymorphisms in the PI3K/AKT/mTOR pathway have been associated with an increased risk of stomach^13^ and bladder^14^ cancers, very few studies have examined the association of this pathway with susceptibility to tuberculosis^15^. The present study systematically screened PTB patients for SNPs in autophagy-related genes of the PI3K/AKT/mTOR pathway and then used a case-control study to evaluate their associations with tuberculosis susceptibility. 

## METHODS

### Study participants

The 401 study participants were recruited from the Han Chinese population presenting to the Shenzhen Nanshan Center for Chronic Disease Control (Nanshan CCDC, 22° N 113° E) from May 2017 to July 2018. This case-control study included treatment-naïve PTB patients whose sputum cultures were positive for *Mtb* and a control group with no clinical, radiological, or bacteriological evidence of TB. The controls were tested using interferon-gamma release assays (IGRA)^16^ and those that tested positive were considered to have LTBI. Positive sputum cultures were subjected to the p-nitrobenzoic acid (PNB) test, and patients whose cultures contained non-tuberculous mycobacteria were excluded. Participants with HBV infection, HIV infection, concomitant chronic obstructive pulmonary disease, or cancer were excluded. 

### Ethics approval

The Ethics Review Committee of the Nanshan CCDC approved this study (ll20170018). All study participants provided written informed consent.

### DNA extraction

Blood samples were collected from the participants using EDTA tubes. The QIAamp® DNA blood mini kit was used to extract genomic DNA, and the extracted DNA was used for subsequent genotyping. 

### SNP selection and genotyping

The Kyoto Encyclopedia of Genes and Genomes (KEGG) pathway database was searched for genes related to the PI3K/AKT or mTOR pathways, and the identified genes were grouped into gene set A (Supplementary Figure 1). Autophagy pathway-related genes in the KEGG database were also identified and compared with autophagy-related genes in the Gene Ontology (GO) database to identify genes common to both databases, which were grouped into gene set B. Pooled gene sets A and B were then compared to identify the genes present in both sets. 

Genes common to both A and B were entered into STRING to generate a protein-protein interactions (PPI) network. The CytoHubba tool from Cytoscape was used to identify the top ten hub genes ranked by twelve algorithms in the PPI network. Hub genes were defined as those with a high correlation in the candidate modules. From the set of hub genes, those identified using at least six algorithms were included.

The tag SNPs of the selected genes were obtained from the 1000 Genomes Project using HaploView 4.2. We then selected SNPs with minor allele frequencies (MAF) ≥ 0.05 and *P* > 0.05 in the Hardy-Weinberg Equilibrium (HWE) test. SNPs in genes with predicted functions and SNPs that had been previously reported in the literature were selected. Genotyping was performed using the Illumina HumanOmniZhongHua-8 BeadChip. Five percent of the samples were chosen for repeat analysis. 

### Statistical analyses

The T-test and chi-square test were used to compare age and sex, respectively. The HWE in controls was evaluated using the SNPassoc package in R 4.0.3^17^. Genetic models (codominant, dominant, and recessive)^18^ were applied to evaluate the impact of genetic variations on the risk of PTB. The genetic risk score (GRS)^19^ was calculated by adding the number of risk alleles (0, 1, or 2) for the PTB-associated SNPs. The generalized multifactor dimensionality reduction (GMDR)^20^ method was applied to investigate SNP-SNP interactions. Age and sex served as covariates in the interaction analyses. Genotyping data were processed using Genome Studio 2.0 and Plink 1.90^21^. IBM SPSS Statistics (version 22.0) was used for the statistical analyses. 

## RESULTS

We included 130 PTB patients and 271 controls with a mean age of 31.18 ± 10.85 and 36.52 ± 12.83, respectively. The controls were divided into two groups: 163 healthy controls (HC) and 108 individuals with LTBI. The age distribution of the PTB patients was significantly different from that of the controls (*P* < 0.05), with more PTB patients in the 15-29 age group and fewer in the 40-89 age group than in either the HC or LTBI groups. This suggests that *Mtb* is more likely to cause diseases in young and middle-aged populations. No significant differences in sex distribution were observed (Table 1).

**TABLE 1:** Characteristics of enrolled subjects.

Characteristics	PTB patients (130)	controls (271)			^P^ PTB patients vs. controls*	^P^ LTBI vs. HC*
		Total	LTBI	HC		
			(IGRA+, 108)	(IGRA-, 163)		
Age, years (mean ± SD)	31.18 ± 10.85	36.52 ± 12.83	40.09 ± 12.42	34.15 ± 12.57	**< 0.001**	**< 0.001**
Age group, years, n (%)						
15-29	76 (58.5)	103(38.0)	26 (24.1)	77 (47.2)	**< 0.001**	**< 0.001**
30-39	34 (26.2)	83 (30.6)	35 (32.4)	48 (29.4)		
40-89	20 (15.3)	85 (31.4)	47 (43.5)	38 (23.4)		
Sex, n (%)						
Male	74 (56.9)	139 (51.3)	54 (50.0)	85 (52.1)	0.290	0.729
Female	56 (43.1)	132 (48.7)	54 (50.0)	78 (47.9)		

* P < 0.05 is in bold.

**FIGURE 1:**
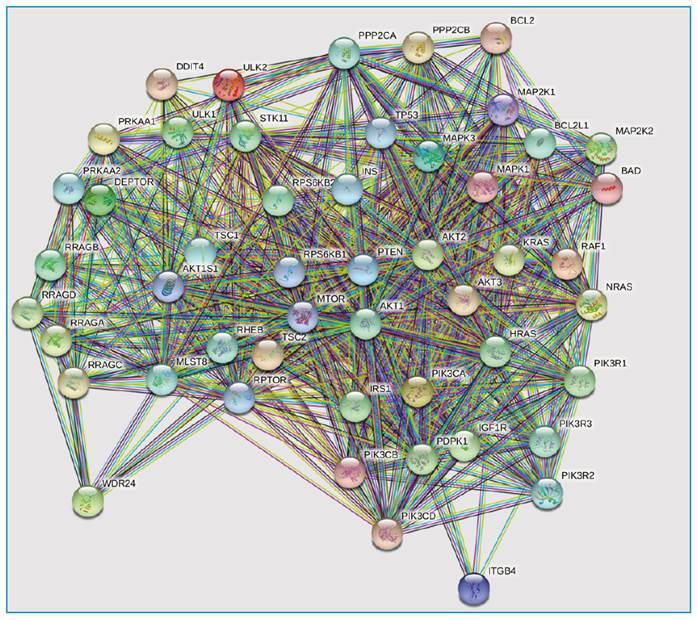
PPI network generated from STRING. The joint genes of gene set A and B (N=50) were put into STRING to generate a protein-protein interactions (PPI) network.

Fifty genes were identified by screening for autophagy-related genes in the PI3K/AKT/mTOR pathway and introduced into STRING to generate a PPI network (Figure 1). Nine hub genes were selected *(AKT1, AKT2, mTOR, PIK3CA, PTEN, RHEB, RPS6KB1, RPTOR,* and *TSC2*), and 30 SNPs found in these genes were analyzed for their associations with PTB susceptibility. The distributions of the allele frequencies for the rs11654508, rs2090204, rs2589144, rs2672897, and rs7503807 SNPs in the *RPTOR* gene and the rs2074969 SNP in the *TSC2* gene had significantly different frequencies in the PTB and control groups (*P* = 0.016, 0.038, 0.001, 0.001, 0.044, and 0.015, respectively, using the chi-square test) (Supplementary Table 1). In the control group, the allele frequencies of all selected SNPs did not deviate from HWE (*P* < 0.05). 

Among the 30 SNPs, one in *AKT1* (rs12432802), five in *RPTOR* (rs11654508, rs12602885, rs2589144, rs2672897, and rs7503807), and one in *TSC2* (rs2074969) were associated with PTB risk in the genetic models (*P* < 0.05) (Table 2). The frequencies of the remaining 23 SNPs were not significantly different (*P* > 0.05) (Supplementary Table 2).

**TABLE 2:** Positive results of genetic model associations between PTB patients and controls.

Gene	SNP	Genotypes	PTB patients	controls^1^	Codominant		Dominant		Recessive	
					(MM/Mm/mm)		(MM/Mm + mm)		(MM + Mm/mm)	
	(M/m)		N (%)		OR (95% CI)	P*	OR (95% CI)	P*	OR (95% CI)	P*
*AKT1*	rs12432802	AA	43(33.1)	69(25.5)	Rf		Rf		Rf	
	A/G	GA	57(43.8)	144(53.1)	0.560(0.323-0.970)	**0.038**	0.651(0.389-1.091)	0.103	1.341(0.754-2.384)	0.318
		GG	30(23.1)	58(21.4)	0.931(0.476-1.822)	0.836				
*RPTOR*	rs11654508	AA	44(33.8)	105(38.7)	Rf		Rf		Rf	
	A/G	GA	51(39.3)	128(47.2)	1.111(0.655-1.884)	0.697	1.366(0.840-2.219)	0.209	2.001(1.110-3.605)	**0.021**
		GG	35(26.9)	38(14.1)	2.116(1.101-4.068)	**0.025**				
	rs12602885	GG	76(58.5)	132(48.7)	Rf		Rf		Rf	
	G/A	AG	49(37.8)	123(45.4)	0.630(0.387-1.025)	0.063	0.611(0.380-0.981)	**0.041**	0.581(0.192-1.757)	0.336
		AA	5(3.8)	16(5.9)	0.467(0.151-1.446)	0.187				
	rs2589144	GG	95(73.1)	152(56.1)	Rf		Rf		Rf	
	G/A	AG	30(23.1)	99(36.5)	0.540(0.317-0.920)	**0.023**	0.527(0.318-0.873)	**0.013**	0.558(0.187-1.664)	0.295
		AA	5(3.8)	20(7.4)	0.459(0.152-1.383)	0.166				
	rs2672897	AA	59(45.4)	87(32.1)	Rf		Rf		Rf	
	A/G	GA	57(43.8)	127(46.9)	0.776(0.465-1.296)	0.332	0.632(0.391-1.021)	0.061	0.410(0.210-0.800)	**0.009**
		GG	14(10.8)	57(21.0)	0.358(0.174-0.737)	**0.005**				
	rs7503807	AA	69(53.0)	119(43.9)	Rf		Rf		Rf	
	A/C	CA	53(40.8)	122(45.0)	0.722(0.441-1.183)	0.196	0.651(0.406-1.045)	0.075	0.458(0.194-1.085)	0.076
		CC	8(6.2)	30(11.1)	0.391(0.160-0.956)	**0.040**				
*TSC2*	rs2074969	GG	100(76.9)	175(64.6)	Rf		Rf		Rf	
	G/A	AG	26(20.0)	83(30.6)	0.442(0.253-0.771)	**0.004**	0.424(0.249-0.719)	**0.001**	0.426(0.129-1.405)	0.161
		AA	4(3.1)	13(4.8)	0.332(0.099-1.110)	0.073				

**M:** Major allele. **m:** minor allele. ^1^ control = LTBI + HC. For all results see Supplementary Table 2. * Adjusted for sex and age (P < 0.05) is in bold.

For the *AKT1* gene, the GA genotype of rs12432802 was less prevalent in the PTB group than in controls (GA vs. AA: OR (95%CI) 0.560 (0.323-0.970); *P* = 0.038). Similarly, the *TSC2* rs2074969 polymorphism was associated with a reduced risk of PTB in the codominant (AG vs. GG: OR (95%CI) 0.442 (0.253-0.771); *P* = 0.004) and dominant models (AG + AA vs. GG: OR (95%CI) 0.424 (0.249-0.719); *P* = 0.001). For the *RPTOR* rs12602885 polymorphism, subjects with the AA + AG genotypes had a relatively lower risk of PTB compared with those carrying the GG genotype in the dominant model (AG + AA vs. GG: OR (95%CI) 0.611 (0.380-0.981); *P* = 0.041). Furthermore, the *RPTOR* rs2589144, rs2672897, and rs7503807 polymorphisms were associated with a significantly reduced risk of PTB in co-dominant models (Table 2). Patients with these three *RPTOR* SNPs had a significantly reduced risk of PTB with the rs2589144 polymorphism in the dominant model (AA + AG vs. GG: OR (95%CI) 0.527 (0.318-0.873); *P* = 0.013) and the rs2672897 polymorphism in the recessive model (GG vs. AA + GA: OR (95%CI) 0.410 (0.210-0.800); *P* = 0.009). In contrast, an increased risk of PTB was associated with the *RPTOR* rs11654508 polymorphism in both the codominant (GG vs. AA: OR (95%CI) 2.116 (1.101-4.068); *P* = 0.025) and recessive models (GG vs. AA + GA: OR (95%CI) 2.001 (1.110-3.605); *P* = 0.021).

When comparing the LTBI and PTB groups, we also found significant associations between the risk of PTB and SNPs in *RPTOR* (rs10871489, rs2090204, rs2589144, and rs2672897) and *TSC2* (rs2074969) (*P* < 0.05) (Supplementary Table 3). For the *RPTOR* (rs10871489, rs2090204, and rs2589144) and *TSC2* (rs2074969) polymorphisms, the Mm genotype was significantly less frequent in the PTB patients with the codominant model (Mm vs. MM: rs10871489, OR (95%CI) 0.535 (0.295-0.970), *P* = 0.039; rs2090204, OR (95%CI) 0.465(0.252-0.858), *P* = 0.014; rs2589144, OR (95%CI) 0.541 (0.293-0.998), *P* = 0.049; rs2074969, OR (95%CI) 0.499 (0.260-0.958), *P* = 0.037, respectively) and also with the dominant model. In addition, when the PTB group was compared with the LTBI group, the *RPTOR* rs2672897 polymorphism was associated with a reduced risk of PTB in the dominant model (GA + GG vs. AA: OR (95%CI) 0.539 (0.302-0.963); *P* = 0.037) (Table 3).

**TABLE 3:** Positive results of genetic model associations between PTB patients and LTBI.

Gene	SNP	Genotypes	PTB patients	LTBI	Codominant		Dominant		Recessive	
					(MM/Mm/mm)		(MM/Mm + mm)		(MM + Mm/mm)	
	(M/m)		N (%)		OR (95% CI)	*P* ^*^	OR (95% CI)	*P* ^*^	OR (95% CI)	*P* ^*^
*RPTOR*	rs10871489	AA	90(69.3)	61(56.5)	Rf		Rf		Rf	
	A/G	GA	35(26.9)	41(38.0)	0.535(0.295-0.970)	**0.039**	0.564(0.319-0.997)	**0.049**	1.020(0.272-3.819)	0.977
		GG	5(3.8)	6(5.5)	0.823(0.216-3.136)	0.775				
	rs2090204	CC	95(73.1)	65(60.2)	Rf		Rf		Rf	
	C/A	AC	30(23.1)	39(36.1)	0.465(0.252-0.858)	**0.014**	0.511(0.284-0.920)	**0.025**	1.433(0.326-6.300)	0.634
		AA	5(3.8)	4(3.7)	1.124(0.251-5.038)	0.878				
	rs2589144	GG	95(73.1)	58(53.7)	Rf		Rf		Rf	
	G/A	AG	30(23.1)	41(38.0)	0.541(0.293-0.998)	**0.049**	0.523(0.293-0.932)	**0.028**	0.543(0.168-1.757)	0.308
		AA	5(3.8)	9(8.3)	0.442(0.134-1.452)	0.178				
	rs2672897	AA	59(45.4)	32(29.6)	Rf		Rf		Rf	
	A/G	GA	57(43.8)	57(52.8)	0.562(0.306-1.032)	0.063	0.539(0.302-0.963)	**0.037**	0.646(0.289-1.442)	0.286
		GG	14(10.8)	19(17.6)	0.462(0.191-1.114)	0.086				
*TSC2*	rs2074969	GG	100(76.9)	75(69.4)	Rf		Rf		Rf	
	G/A	AG	26(20.0)	30(27.8)	0.499(0.260-0.958)	**0.037**	0.512(0.273-0.960)	**0.037**	0.796(0.155-4.084)	0.785
		AA	4(3.1)	3(2.8)	0.648(0.124-3.395)	0.608				

M: Major allele. m: minor allele. For all results see Supplementary Table 3. * Adjusted for sex and age (P < 0.05) is in bold.

We compared the HC and LTBI groups and found that only *AKT1*(rs11848899) and *RHEB* (rs3789817) were associated with a reduced risk of LTBI (Supplementary Table 4). For the *AKT1* rs11848899 polymorphism, the LTBI group had a significantly lower frequency of the AC genotype (AC vs. CC: OR (95%CI) 0.558 (0.318-0.979); *P* = 0.042) and the AC + AA genotypes (AC + AA vs. CC: OR (95%CI) 0.539 (0.312-0.931); *P* = 0.027) than the CC genotype. For the *RHEB* rs3789817 polymorphism, the AG + AA genotypes were also significantly less common in the LTBI group than in the HC group.

The 11 SNPs (*AKT*: rs12432802 and rs11848899; *RHEB*: rs3789817; *RPTOR*: rs11654508, rs12602885, rs2589144, rs2672897, rs2090204, rs10871489, and rs7503807; *TSC2*: rs2074969) with significantly different group frequencies were included in the calculation of GRS. Individual genetic risk scores, ranging from a minimum of 0 to a maximum of 16, were combined into subgroups of 0-5, 6-10, and 11-16 (Table 4). The mean GRS was lower in the PTB group than in the control group (mean GRS:5.88 vs. 6.94, *P* < 0.001), and there was a positive trend (*P* trend = 0.020). Compared to individuals with a GRS of 0-5, those with a higher GRS were less likely to have PTB (GRS 6-10, OR (95%CI) 0.590 (0.374-0.931), *P* = 0.023; GRS 11-16, OR (95%CI) 0.381 (0.160-0.906), *P* = 0.029).

**TABLE 4:** The relationship between the GRS and tuberculosis susceptibility.

GRS ^a^	PTB patients (130)	controls (271)	PTB patients vs. controls		
			T or OR (95% CI)	P*	***P* trend**
Mean ± SD	5.88 ± 2.597	6.94 ± 2.822	3.613	**< 0.001**	
Subgroup, n (%)					
0-5	59 (45.4)	86 (31.7)	1.00		**0.020**
6-10	63 (48.5)	156 (57.6)	0.590 (0.374-0.931)	**0.023**	
11-16	8 (6.1)	29 (10.7)	0.381 (0.160-0.906)	**0.029**	

**GRS:** genetic risk score. ^a^ A total of 11 SNPS (*AKT*: rs12432802 and rs11848899; *RHEB*: rs3789817; *RPTOR*: rs11654508, rs12602885, rs2589144, rs2672897, rs2090204, rs10871489, and rs7503807; *TSC2*: rs2074969) were included in the calculation of GRS. * Adjusted for sex and age (*P* < 0.05) is in bold.

We then analyzed SNP-SNP interactions with or without a diagnosis of PTB as the outcome of GMDR. There was a significant difference in only one SNP (rs11848899) in the interaction model (*P* = 0.0107), with a testing accuracy of 50.45% (Supplementary Table 5). No significant differences were found in the interaction models with two or more SNPs.

## DISCUSSION

This study investigated the associations of 30 SNPs in autophagy-related genes of the PI3K/AKT/mTOR pathway with the risk of PTB in a Han Chinese population. The results revealed that *AKT1* (rs12432802), *RPTOR* (rs12602885, rs2589144, rs2672897, rs7503807), and *TSC2* (rs2074969) polymorphisms were associated with a significantly lower risk of developing PTB, whereas the *RPTOR* (rs11654508) polymorphism was associated with a significantly higher risk of PTB. The results also showed that a lower GRS based on the significant SNPs identified in our study was associated with a higher risk of PTB.

*AKT1, RPTOR,* and *TSC2* were identified as hub genes in our study. AKT1 activation contributes to the transition of macrophages into alternatively activated phenotypes (M2)^22^. The transition of host macrophages from classically activated phenotypes (M1) to M2 phenotypes is one way by which *Mtb* can avoid hostile environments^23^. Among the five *AKT1* SNPs, the *AKT1* rs12432802 polymorphism was associated with a decreased risk of PTB, whereas the rs11848899 polymorphism was more frequent in the LTBI group. We found no previous publications on these two SNPs. Wang et al.^24^ found that the *AKT1* rs3730358 and rs1130233 polymorphisms were associated with susceptibility to PTB, but the association with rs1130233 was not confirmed in our study, perhaps due to differences in allele frequencies between Han populations in northern and southern China or inadequate sample sizes in either or both studies. *AKT1* polymorphisms were associated with clinical outcomes in patients with lung^25^ and gastric cancers^26^; however, a separate study found that *AKT1* polymorphisms are rare and not valuable as biomarkers for bladder cancer management^27^. 

During immunization or infection, RPTOR is involved in initiating T-follicular regulatory cell differentiation^28^, and hypomethylation of the *RPTOR* gene is thought to be associated with TB involving the pleura^29^. Of the ten *RPTOR* SNPs included in our study, five (rs12602885, rs2589144, rs2672897, rs7503807, and rs11654508) were significantly associated with the risk of TB. The rs12602885 polymorphism has been extensively studied for its association with cancer risk; however, a meta-analysis found no association with cancer in the Chinese population^30^. The association of the rs12602885 with a decreased risk of PTB has not been previously reported. Polymorphisms in the *RPTOR* gene have been associated with an increased risk of pancreatic cancer^31^ and obesity^32^, but the studies of these associations have been conflicting and inconclusive.

The TSC2 (tuberous sclerosis 2) gene is essential for maintaining macrophage quiescence to prevent the development of granulomatous disease via an mTOR-dependent pathway^33^. *Mtb* infections classically cause granulomas within the lungs, and the early formation of granulomas is thought to favor the development of LTBI^34^ rather than PTB. We found that the *TSC2* rs2074969 polymorphism was associated with decreased susceptibility to PTB compared to the LTBI group. However, when the LTBI group was compared to the HC group, the association was not significant. Similar to a previous study using TSC2 conditional knockout mice^33^, we suggest that *TSC2* may be involved in the development of TB and therefore *TSC2* polymorphisms could be biomarkers for disease progression. Mutations in the *TSC1* and *TSC2* genes have been reported as the genetic basis for the tuberous sclerosis disease complex, and *TSC2* gene variation has been associated with status epilepticus in Chinese children^35^. 

Ras homologue enriched in brain (RHEB) is a target gene of microRNA-155 and contributes to a reduction in the survival of intracellular mycobacteria in macrophages^36^. *RHEB* gene polymorphisms may be associated with susceptibility to *Mtb* infection when compared with the HC group but not when compared with the PTB group. The *RHEB* rs1109089 polymorphism has been associated with suicide in a historically high-risk isolated population from Northeast India^37^; however, there are few other published studies on *RHEB* gene polymorphisms. 

Using a simple GRS based on our SNPs with statistically significant associations, we found that lower GRS scores were associated with an increased risk of PTB compared with controls, but there was no association of GRS scores with LTBI. Our GMDR analysis revealed no obvious interactions among the 11 significantly associated SNPs, and the highest testing accuracy was 50.45%. However, the failure to identify interactions could be due to the limited number of loci examined and the relatively small sample size.

This study had some limitations. First, we included only individuals from Nanshan's Han Chinese population, and the number of participants may not have been sufficiently large to detect small effects. Therefore, the identified associations should be confirmed in larger studies involving different ethnicities. Second, healthy controls were evaluated for LTBI using IGRA but not the Mantoux tuberculin skin test (TST). Although both IGRA and TST are recommended by the WHO^16^, we employed IGRA because all participants were vaccinated with Bacillus Calmette-Guerin (BCG). Third, only a limited number of SNPs were identified. The key role of the PI3K/AKT/mTOR pathway in PTB pathology suggests that studies identifying additional risk loci and haplotypes in this pathway are worthwhile.

In summary, this study of PTB and control groups drawn from the Han Chinese population of Nanshan, Shenzhen found significant associations between the risk of developing PTB and polymorphisms in genes related to the PI3K/AKT/mTOR pathway. Based on the known functions of the encoded proteins, we suggest that polymorphisms in *AKT1, RPTOR,* and *TSC2* may alter PTB susceptibility by modifying autophagy-mediated immune responses to *Mtb*. Additionally, a reduction in the GRS was associated with an increased risk of PTB. Studies with larger sample sizes and ethnically diverse populations are required to confirm these associations.
