# Supplementary figures and images for: Association of PI3K/AKT/mTOR pathway autophagy-related gene polymorphisms with pulmonary tuberculosis susceptibility in a Chinese population
Source: Rev Soc Bras Med Trop. 2023 Jul 24;56:e0104-2023. doi: 10.1590/0037-8682-0104-2023 (PMC10367219; doi:10.1590/0037-8682-0104-2023)

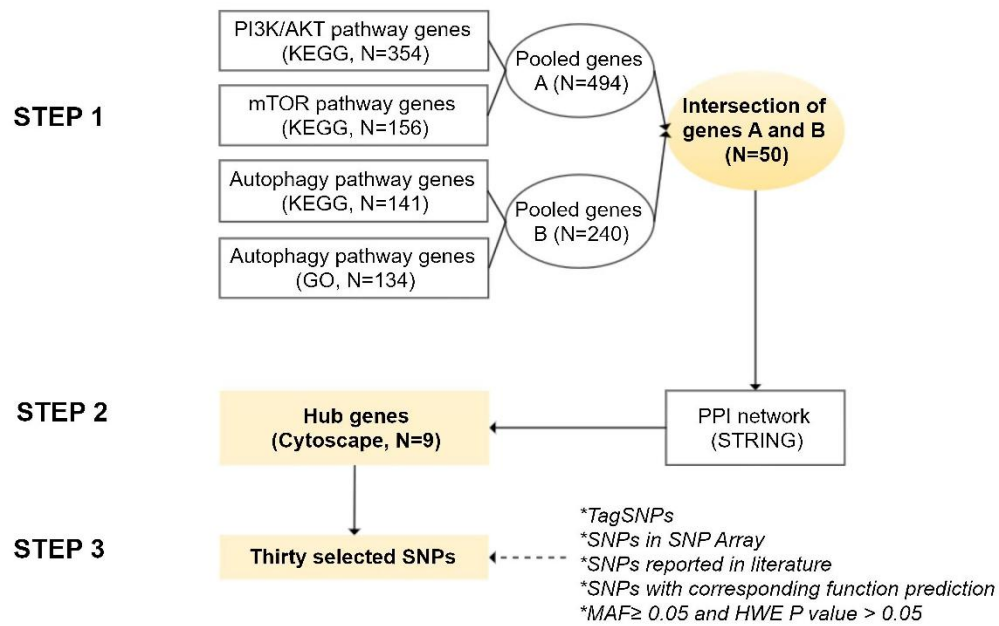**SUPPLEMENTARY FIGURE 1:** The workflow of SNPs selection.

Supplement: Supplementary file 1 [file 1678-9849-rsbmt-56-e0104-2023-supp1.pdf]
