# Supplementary material for: Association of PI3K/AKT/mTOR pathway autophagy-related gene polymorphisms with pulmonary tuberculosis susceptibility in a Chinese population
Source: Rev Soc Bras Med Trop. 2023 Jul 24;56:e0104-2023. doi: 10.1590/0037-8682-0104-2023 (PMC10367219; doi:10.1590/0037-8682-0104-2023)
Supplement: Supplementary file 2 [file 1678-9849-rsbmt-56-e0104-2023-supp2.pdf]

SUPPLEMENTARY TABLE 1: Distributions of the allele for 30 SNPs in the nine genes.

Gene	SNP	Major/minor Allele	MAF in PTB patients	MAF in controls[1]	c^2	P^*	HWE-*P* [a]
AKT1	rs1130233	A/G	0.365	0.410	1.437	0.231	0.209
AKT1	rs11848899	C/A	0.154	0.190	1.531	0.216	0.691
AKT1	rs12432802	A/G	0.450	0.480	0.622	0.430	0.331
AKT1	rs2494738	A/G	0.438	0.459	0.324	0.569	0.465
AKT1	rs2494743	G/A	0.296	0.282	0.165	0.684	0.882
AKT2	rs1991823	A/G	0.415	0.382	0.825	0.364	0.071
AKT2	rs4803320	G/A	0.350	0.339	0.096	0.756	0.135
mTOR	rs12122605	G/A	0.223	0.234	0.125	0.724	1.000
mTOR	rs2536	A/G	0.088	0.101	0.339	0.560	0.745
mTOR	rs3806317	A/G	0.128	0.131	0.011	0.916	0.420
PIK3CA	rs1607237	G/A	0.327	0.275	2.301	0.129	1.000
PIK3CA	rs2677760	G/A	0.238	0.225	0.178	0.673	0.226
PTEN	rs2299939	C/A	0.185	0.188	0.015	0.903	1.000
PTEN	rs741804	A/C	0.165	0.159	0.049	0.825	1.000
RHEB	rs2299962	G/A	0.346	0.308	1.167	0.280	0.479
RHEB	rs3789817	G/A	0.438	0.454	0.165	0.685	0.541
RHEB	rs6972955	A/C	0.469	0.448	0.315	0.575	0.141
RPS6KB1	rs180515	A/G	0.446	0.457	0.090	0.765	0.903
RPS6KB1	rs180519	A/G	0.473	0.428	1.444	0.229	0.456
RPTOR	rs10871489	A/G	0.173	0.232	3.695	0.055	0.524
RPTOR	rs11651587	A/G	0.365	0.380	0.162	0.688	0.798
RPTOR	rs11654508	A/G	0.465	0.376	5.775	**0.016**	1.000
RPTOR	rs12602885	G/A	0.227	0.286	3.132	0.077	0.076
RPTOR	rs2090204	C/A	0.154	0.216	4.293	**0.038**	0.859
RPTOR	rs2589144	G/A	0.154	0.256	10.671	**0.001**	0.524
RPTOR	rs2672897	A/G	0.327	0.445	10.094	**0.001**	0.392
RPTOR	rs7209040	G/A	0.300	0.306	0.033	0.857	0.886
RPTOR	rs7224758	G/A	0.173	0.131	2.515	0.113	0.791
RPTOR	rs7503807	A/C	0.265	0.336	4.051	**0.044**	1.000
TSC2	rs2074969	G/A	0.131	0.201	5.933	**0.015**	0.450

SNP: single nucleotide polymorphisms. PTB: pulmonary tuberculosis. MAF: Minor allele frequency. OR: odds ratio. 95% CI: 95% confidence interval.

[1] control = LTBI + HC. (LTBI: latent tuberculosis infection, HC: health control).

[a] HWE-P: Hardy-Weinberg equilibrium-P value in controls. * ＜0.05 is in bold.
