# Supplementary material for: Association of PI3K/AKT/mTOR pathway autophagy-related gene polymorphisms with pulmonary tuberculosis susceptibility in a Chinese population
Source: Rev Soc Bras Med Trop. 2023 Jul 24;56:e0104-2023. doi: 10.1590/0037-8682-0104-2023 (PMC10367219; doi:10.1590/0037-8682-0104-2023)
Supplement: Supplementary file 3 [file 1678-9849-rsbmt-56-e0104-2023-supp3.pdf]

SUPPLEMENTARY TABLE 2: Genetic model associations between PTB and controls for selected SNPs.

Gene	SNP (m/M)	Genotypes	PTB patients	controls[1]	Codominant (MM/Mm/mm)		Dominant (MM/Mm + mm)		Recessive (MM + Mm/mm)	
			N (%)		OR (95% CI)	P^*	OR (95% CI)	P^*	OR (95% CI)	P^*
AKT1	rs1130233	AA	53(40.8)	89(32.8)	Rf		Rf		Rf	
	G/A	GA	59(45.4)	142(52.4)	0.733(0.441-1.216)	0.229	0.756(0.467-1.224)	0.256	1.011(0.515-1.985)	0.975
		GG	18(13.8)	40(14.8)	0.847(0.407-1.763)	0.656				
	rs11848899	CC	92(70.8)	175(65.0)	Rf		Rf		Rf	
	A/C	AC	36(27.7)	86(32.0)	0.676(0.406-1.125)	0.132	0.645(0.392-1.060)	0.084	0.398(0.078-2.036)	0.269
		AA	2(1.5)	8(3.0)	0.352(0.068-1.812)	0.212				
	rs12432802	AA	43(33.1)	69(25.5)	Rf		Rf		Rf	
	G/A	GA	57(43.8)	144(53.1)	0.560(0.323-0.970)	**0.038**	0.651(0.389-1.091)	0.103	1.341(0.754-2.384)	0.318
		GG	30(23.1)	58(21.4)	0.931(0.476-1.822)	0.836				
	rs2494738	AA	42(32.6)	76(28.0)	Rf		Rf		Rf	
	G/A	GA	61(47.3)	141(52.1)	0.745(0.435-1.276)	0.283	0.793(0.476-1.319)	0.371	1.125(0.624-2.027)	0.696
		GG	26(20.1)	54(19.9)	0.934(0.473-1.842)	0.843				
	rs2494743	GG	68(52.3)	140(51.7)	Rf		Rf		Rf	
	A/G	AG	47(36.2)	109(40.2)	0.795(0.485-1.303)	0.363	0.902(0.566-1.436)	0.663	1.739(0.764-3.960)	0.187
		AA	15(11.5)	22(8.1)	1.573(0.672-3.681)	0.296				
AKT2	rs1991823	AA	41(31.6)	96(35.4)	Rf		Rf		Rf	
	G/A	GA	70(53.8)	143(52.8)	1.034(0.616-1.735)	0.899	1.079(0.656-1.777)	0.764	1.262(0.635-2.510)	0.507
		GG	19(14.6)	32(11.8)	1.289(0.603-2.758)	0.513				
	rs4803320	GG	54(41.5)	112(41.5)	Rf		Rf		Rf	
	A/G	AG	61(46.9)	133(49.3)	0.872(0.532-1.428)	0.586	0.929(0.579-1.492)	0.761	1.367(0.629-2.967)	0.430
		AA	15(11.6)	25(9.3)	1.268(0.558-2.880)	0.570				
mTOR	rs12122605	GG	78(60.0)	159(58.7)	Rf		Rf		Rf	
	A/G	AG	46(35.4)	97(35.8)	1.006(0.614-1.647)	0.982	0.987(0.614-1.585)	0.955	0.854(0.286-2.551)	0.777
		AA	6(4.6)	15(5.5)	0.856(0.282-2.595)	0.783				
	rs2536	AA	108(83.1)	219(80.8)	Rf		Rf		Rf	

SUPPLEMENTARY TABLE 2: Genetic model associations between PTB and controls for selected SNPs.

Gene	SNP (m/M)	Genotypes	PTB patients	controls[1]	Codominant (MM/Mm/mm)		Dominant (MM/Mm + mm)		Recessive (MM + Mm/mm)	
			N (%)		OR (95% CI)	P^*	OR (95% CI)	P^*	OR (95% CI)	P^*
	G/A	GA	21(16.2)	49(18.1)	1.017(0.540-1.914)	0.958	0.991(0.535-1.838)	0.978	0.646(0.057-7.373)	0.725
		GG	1(0.8)	3(1.1)	0.648(0.057-7.410)	0.727				
	rs3806317	AA	98(76.0)	204(76.1)	Rf		Rf		Rf	
	G/A	GA	29(22.4)	58(21.6)	0.919(0.527-1.600)	0.765	0.926(0.538-1.593)	0.781	1.073(0.148-7.777)	0.944
		GG	2(1.6)	6(2.3)	1.052(0.145-7.658)	0.960				
PIK3CA	rs1607237	GG	58(44.6)	142(52.4)	Rf		Rf		Rf	
	A/G	AG	59(45.4)	109(40.2)	1.281(0.784-2.093)	0.323	1.333(0.833-2.132)	0.231	1.454(0.629-3.359)	0.381
		AA	13(10.0)	20(7.4)	1.639(0.686-3.921)	0.266				
	rs2677760	GG	76(58.5)	159(58.7)	Rf		Rf		Rf	
	A/G	AG	46(35.4)	102(37.6)	0.879(0.538-1.437)	0.608	0.962(0.599-1.547)	0.874	2.237(0.698-7.161)	0.175
		AA	8(6.1)	10(3.7)	2.121(0.651-6.908)	0.212				
PTEN	rs2299939	CC	88(67.7)	178(65.7)	Rf		Rf		Rf	
	A/C	AC	36(27.7)	84(31.0)	0.785(0.468-1.316)	0.358	0.813(0.496-1.334)	0.413	1.142(0.356-3.658)	0.823
		AA	6(4.6)	9(3.3)	1.055(0.325-3.422)	0.929				
	rs741804	AA	92(70.8)	191(70.7)	Rf		Rf		Rf	
	C/A	CA	33(25.4)	72(26.7)	0.949(0.555-1.623)	0.848	1.028(0.614-1.722)	0.916	2.203(0.508-9.556)	0.292
		CC	5(3.8)	7(2.6)	2.174(0.498-9.491)	0.302				
RHEB	rs2299962	GG	56(43.1)	127(46.9)	Rf		Rf		Rf	
	A/G	AG	58(44.6)	121(44.6)	1.076(0.656-1.765)	0.771	1.159(0.723-1.857)	0.540	1.568(0.706-3.480)	0.269
		AA	16(12.3)	23(8.5)	1.626(0.706-3.744)	0.253				
	rs3789817	GG	42(32.3)	83(30.7)	Rf		Rf		Rf	
	A/G	AG	62(47.7)	129(47.8)	0.783(0.454-1.349)	0.378	0.754(0.452-1.257)	0.279	0.807(0.457-1.427)	0.462
		AA	26(20.0)	58(21.5)	0.694(0.358-1.345)	0.279				
	rs6972955	AA	37(28.5)	76(28.1)	Rf		Rf		Rf	
	C/A	CA	64(49.2)	146(54.1)	1.004(0.585-1.722)	0.989	1.122(0.673-1.870)	0.659	1.507(0.827-2.746)	0.180

SUPPLEMENTARY TABLE 2: Genetic model associations between PTB and controls for selected SNPs.

Gene	SNP (m/M)	Genotypes	PTB patients	controls[1]	Codominant (MM/Mm/mm)		Dominant (MM/Mm + mm)		Recessive (MM + Mm/mm)	
			N (%)		OR (95% CI)	P^*	OR (95% CI)	P^*	OR (95% CI)	P^*
		CC	29(22.3)	48(17.8)	1.510(0.759-3.005)	0.240				
RPS6KB1	rs180515	AA	42(32.3)	80(29.6)	Rf		Rf		Rf	
	G/A	GA	60(46.2)	133(49.3)	0.799(0.467-1.370)	0.415	0.832(0.502-1.380)	0.477	1.049(0.592-1.858)	0.870
		GG	28(21.5)	57(21.1)	0.914(0.472-1.769)	0.789				
	rs180519	AA	42(32.3)	92(33.9)	Rf		Rf		Rf	
	G/A	GA	53(40.8)	126(46.5)	1.060(0.621-1.812)	0.830	1.226(0.751-2.002)	0.415	1.550(0.891-2.697)	0.120
		GG	35(26.9)	53(19.6)	1.601(0.855-2.999)	0.141				
RPTOR	rs10871489	AA	90(69.3)	161(59.4)	Rf		Rf		Rf	
	G/A	GA	35(26.9)	94(34.7)	0.731(0.435-1.227)	0.236	0.703(0.429-1.152)	0.162	0.614(0.203-1.857)	0.387
		GG	5(3.8)	16(5.9)	0.555(0.181-1.699)	0.302				
	rs11651587	AA	49(37.7)	103(38.0)	Rf		Rf		Rf	
	G/A	GA	67(51.5)	130(48.0)	1.150(0.695-1.901)	0.587	1.046(0.647-1.690)	0.854	0.675(0.333-1.369)	0.276
		GG	14(10.8)	38(14.0)	0.730(0.341-1.564)	0.418				
	rs11654508	AA	44(33.8)	105(38.7)	Rf		Rf		Rf	
	G/A	GA	51(39.3)	128(47.2)	1.111(0.655-1.884)	0.697	1.366(0.840-2.219)	0.209	2.001(1.110-3.605)	**0.021**
		GG	35(26.9)	38(14.1)	2.116(1.101-4.068)	**0.025**				
	rs12602885	GG	76(58.5)	132(48.7)	Rf		Rf		Rf	
	A/G	AG	49(37.8)	123(45.4)	0.630(0.387-1.025)	0.063	0.611(0.380-0.981)	**0.041**	0.581(0.192-1.757)	0.336
		AA	5(3.8)	16(5.9)	0.467(0.151-1.446)	0.187				
	rs2090204	CC	95(73.1)	167(61.6)	Rf		Rf		Rf	
	A/C	AC	30(23.1)	91(33.6)	0.610(0.356-1.047)	0.073	0.612(0.367-1.021)	0.060	0.727(0.235-2.250)	0.581
		AA	5(3.8)	13(4.8)	0.624(0.199-1.954)	0.418				
	rs2589144	GG	95(73.1)	152(56.1)	Rf		Rf		Rf	
	A/G	AG	30(23.1)	99(36.5)	0.540(0.317-0.920)	**0.023**	0.527(0.318-0.873)	**0.013**	0.558(0.187-1.664)	0.295
		AA	5(3.8)	20(7.4)	0.459(0.152-1.383)	0.166				

SUPPLEMENTARY TABLE 2: Genetic model associations between PTB and controls for selected SNPs.

Gene	SNP (m/M)	Genotypes	PTB patients	controls[1]	Codominant (MM/Mm/mm)		Dominant (MM/Mm + mm)		Recessive (MM + Mm/mm)	
			N (%)		OR (95% CI)	*P*[*]	OR (95% CI)	*P*[*]	OR (95% CI)	*P*[*]
	rs2672897	AA	59(45.4)	87(32.1)	Rf		Rf		Rf	
	G/A	GA	57(43.8)	127(46.9)	0.776(0.465-1.296)	0.332	0.632(0.391-1.021)	0.061	0.410(0.210-0.800)	**0.009**
		GG	14(10.8)	57(21.0)	0.358(0.174-0.737)	**0.005**				
	rs7209040	GG	62(47.7)	131(48.3)	Rf		Rf		Rf	
	A/G	AG	58(44.6)	114(42.1)	0.932(0.573-1.518)	0.778	0.895(0.561-1.428)	0.641	0.750(0.327-1.720)	0.497
		AA	10(7.7)	26(9.6)	0.724(0.305-1.720)	0.465				
	rs7224758	GG	89(68.5)	205(75.6)	Rf		Rf		Rf	
	A/G	AG	37(28.4)	61(22.6)	1.485(0.866-2.546)	0.151	1.549(0.917-2.614)	0.102	2.366(0.424-13.195)	0.326
		AA	4(3.1)	5(1.8)	2.614(0.466-14.661)	0.275				
	rs7503807	AA	69(53.0)	119(43.9)	Rf		Rf		Rf	
	C/A	CA	53(40.8)	122(45.0)	0.722(0.441-1.183)	0.196	0.651(0.406-1.045)	0.075	0.458(0.194-1.085)	0.076
		CC	8(6.2)	30(11.1)	0.391(0.160-0.956)	**0.040**				
TSC2	rs2074969	GG	100(76.9)	175(64.6)	Rf		Rf		Rf	
	A/G	AG	26(20.0)	83(30.6)	0.442(0.253-0.771)	**0.004**	0.424(0.249-0.719)	**0.001**	0.426(0.129-1.405)	0.161
		AA	4(3.1)	13(4.8)	0.332(0.099-1.110)	0.073				

SNP: single nucleotide polymorphisms. PTB: pulmonary tuberculosis. 95% CI:95% confidence interval. OR: odds ratio. Rf: Reference. M: Major allele. m: minor allele. MM: homozygote of major allele. Mm: heterozygote. mm: homozygote of minor allele.

[1] control = LTBI + HC. (LTBI: latent tuberculosis infection, HC: health control).

[*] Adjusted by sex and age, ＜0.05 is in bold.
