# Supplementary material for: Association of PI3K/AKT/mTOR pathway autophagy-related gene polymorphisms with pulmonary tuberculosis susceptibility in a Chinese population
Source: Rev Soc Bras Med Trop. 2023 Jul 24;56:e0104-2023. doi: 10.1590/0037-8682-0104-2023 (PMC10367219; doi:10.1590/0037-8682-0104-2023)
Supplement: Supplementary file 4 [file 1678-9849-rsbmt-56-e0104-2023-supp4.pdf]

SUPPLEMENTARY TABLE 3: Genetic model associations between PTB and LTBI for selected SNPs.

Gene	SNP (m/M)	Genotypes	PTB patients	LTBI	Codominant (MM/Mm/mm)		Dominant (MM/Mm + mm)		Recessive (MM + Mm/mm)	
			N (%)		OR (95% CI)	P^*	OR (95% CI)	P^*	OR (95% CI)	P^*
AKT1	rs1130233	AA	53(40.8)	34(31.5)	Rf					
	G/A	GA	59(45.4)	58(53.7)	0.654(0.358-1.195)	0.167	0.689(0.388-1.224)	0.204	1.062(0.477-2.361)	0.883
		GG	18(13.8)	16(14.8)	0.831(0.347-1.990)	0.678				
	rs11848899	CC	92(70.8)	78(72.2)						
	A/C	AC	36(27.7)	28(25.9)	1.065(0.571-1.987)	0.842	1.048(0.569-1.930)	0.879	0.781(0.086-7.117)	0.826
		AA	2(1.5)	2(1.9)	0.795(0.087-7.286)	0.839				
	rs12432802	AA	43(33.1)	29(26.8)						
	G/A	GA	57(43.8)	53(49.1)	0.649(0.340-1.237)	0.649	0.739(0.406-1.348)	0.324	1.251(0.643-2.434)	0.510
		GG	30(23.1)	26(24.1)	0.966(0.447-2.091)	0.966				
	rs2494738	AA	42(32.6)	32(29.6)						
	G/A	GA	61(47.3)	53(49.1)	0.926(0.494-1.736)	0.810	0.976(0.540-1.764)	0.936	1.165(0.582-2.330)	0.666
		GG	26(20.1)	23(21.3)	1.112(0.504-2.453)	0.793				
	rs2494743	GG	68(52.3)	59(54.6)						
	A/G	AG	47(36.2)	38(35.2)	1.282(0.702-2.340)	0.418	1.235(0.709-2.150)	0.456	0.997(0.412-2.413)	0.995
		AA	15(11.5)	11(10.2)	1.097(0.440-2.737)	0.843				
AKT2	rs1991823	AA	41(31.6)	41(38.0)						
	G/A	GA	70(53.8)	54(50.0)	1.225(0.669-2.243)	0.510	1.220(0.683-2.177)	0.502	1.059(0.475-2.360)	0.889
		GG	19(14.6)	13(12.0)	1.198(0.496-2.892)	0.688				
	rs4803320	GG	54(41.5)	46(43.0)						
	A/G	AG	61(46.9)	50(46.7)	0.977(0.544-1.755)	0.937	0.979(0.560-1.713)	0.941	1.002(0.416-2.411)	0.997
		AA	15(11.6)	11(10.3)	0.989(0.389-2.512)	0.982				
mTOR	rs12122605	GG	78(60.0)	62(57.4)						
	A/G	AG	46(35.4)	39(36.1)	0.946(0.528-1.694)	0.852	0.867(0.497-1.514)	0.616	0.498(0.150-1.650)	0.254
		AA	6(4.6)	7(6.5)	0.487(0.144-1.650)	0.248				
	rs2536	AA	108(83.1)	84(77.8)						

SUPPLEMENTARY TABLE 3: Genetic model associations between PTB and LTBI for selected SNPs.

Gene	SNP (m/M)	Genotypes	PTB patients	LTBI	Codominant (MM/Mm/mm)		Dominant (MM/Mm + mm)		Recessive (MM + Mm/mm)	
			N (%)		OR (95% CI)	P^*	OR (95% CI)	P^*	OR (95% CI)	P^*
	G/A	GA	21(16.2)	23(21.3)	0.683(0.340-1.374)	0.285	0.671(0.338-1.331)	0.254	0.504(0.031-8.267)	0.631
		GG	1(0.8)	1(0.9)	0.468(0.028-7.715)	0.596				
	rs3806317	AA	98(76.0)	84(78.5)						
	G/A	GA	29(22.4)	19(17.8)	1.378(0.684-2.778)	0.370	1.156(0.598-2.234)	0.667	0.292(0.049-1.751)	0.178
		GG	2(1.6)	4(3.7)	0.310(0.051-1.871)	0.202				
PIK3CA	rs1607237	GG	58(44.6)	60(55.6)						
	A/G	AG	59(45.4)	40(37.0)	1.535(0.858-2.744)	0.149	1.651(0.947-2.877)	0.077	1.977(0.706-5.540)	0.195
		AA	13(10.0)	8(7.4)	2.410(0.830-6.998)	0.106				
	rs2677760	GG	76(58.5)	66(61.1)						
	A/G	AG	46(35.4)	37(34.3)	1.003(0.557-1.807)	0.991	1.103(0.627-1.941)	0.734	2.155(0.588-7.899)	0.247
		AA	8(6.1)	5(4.6)	2.158(0.577-8.064)	0.253				
PTEN	rs2299939	CC	88(67.7)	73(67.6)						
	A/C	AC	36(27.7)	32(29.6)	0.693(0.373-1.289)	0.247	0.769(0.423-1.396)	0.388	2.011(0.415-9.749)	0.386
		AA	6(4.6)	3(2.8)	1.820(0.367-9.021)	0.463				
	rs741804	AA	92(70.8)	74(68.5)						
	C/A	CA	33(25.4)	30(27.8)	0.783(0.416-1.464)	0.443	0.807(0.444-1.467)	0.482	1.073(0.252-4.568)	0.924
		CC	5(3.8)	4(3.7)	1.004(0.233-4.324)	0.995				
RHEB	rs2299962	GG	56(43.1)	51(47.2)						
	A/G	AG	58(44.6)	46(42.6)	1.304(0.725-2.344)	0.376	1.224(0.705-2.127)	0.473	0.846(0.351-2.037)	0.708
		AA	16(12.3)	11(10.2)	0.959(0.382-2.413)	0.930				
	rs3789817	GG	42(32.3)	40(37.4)						
	A/G	AG	62(47.7)	47(43.9)	1.328(0.714-2.467)	0.370	1.236(0.695-2.201)	0.471	0.889(0.441-1.791)	0.742
		AA	26(20.0)	20(18.7)	1.043(0.477-2.280)	0.916				
	rs6972955	AA	37(28.5)	26(24.3)						
	C/A	CA	64(49.2)	58(54.2)	1.086(0.555-2.126)	0.810	1.008(0.537-1.891)	0.980	0.820(0.423-1.589)	0.557

SUPPLEMENTARY TABLE 3: Genetic model associations between PTB and LTBI for selected SNPs.

Gene	SNP (m/M)	Genotypes	PTB patients	LTBI	Codominant (MM/Mm/mm)		Dominant (MM/Mm + mm)		Recessive (MM + Mm/mm)	
			N (%)		OR (95% CI)	P^{*}	OR (95% CI)	P^{*}	OR (95% CI)	P^{*}
		CC	29(22.3)	23(21.5)	0.864(0.393-1.900)	0.717				
RPS6KB1	rs180515	AA	42(32.3)	34(31.5)						
	G/A	GA	60(46.2)	50(46.3)	0.860(0.454-1.628)	0.643	0.834(0.460-1.513)	0.550	0.855(0.441-1.657)	0.643
		GG	28(21.5)	24(22.2)	0.781(0.363-1.680)	0.528				
	rs180519	AA	42(32.3)	32(29.6)						
	G/A	GA	53(40.8)	55(50.9)	0.764(0.403-1.449)	0.409	0.956(0.527-1.732)	0.881	1.773(0.905-3.473)	0.095
		GG	35(26.9)	21(19.5)	1.509(0.696-3.272)	0.297				
RPTOR	rs10871489	AA	90(69.3)	61(56.5)						
	G/A	GA	35(26.9)	41(38.0)	0.535(0.295-0.970)	**0.039**	0.564(0.319-0.997)	**0.049**	1.020(0.272-3.819)	0.977
		GG	5(3.8)	6(5.5)	0.823(0.216-3.136)	0.775				
	rs11651587	AA	49(37.7)	39(36.1)						
	G/A	GA	67(51.5)	56(51.9)	0.985(0.546-1.778)	0.961	1.029(0.582-1.818)	0.923	1.283(0.525-3.138)	0.585
		GG	14(10.8)	13(12.0)	1.272(0.487-3.322)	0.623				
	rs11654508	AA	44(33.8)	36(33.3)						
	G/A	GA	51(39.3)	58(53.7)	0.654(0.351-1.220)	0.182	0.850(0.474-1.523)	0.585	2.051(0.996-4.226)	0.051
		GG	35(26.9)	14(13.0)	1.606(0.715-3.609)	0.251				
	rs12602885	GG	76(58.5)	56(51.8)						
	A/G	AG	49(37.8)	46(42.6)	0.764(0.430-1.359)	0.360	0.728(0.416-1.274)	0.267	0.534(0.145-1.965)	0.346
		AA	5(3.8)	6(5.6)	0.474(0.126-1.787)	0.270				
	rs2090204	CC	95(73.1)	65(60.2)						
	A/C	AC	30(23.1)	39(36.1)	0.465(0.252-0.858)	**0.014**	0.511(0.284-0.920)	**0.025**	1.433(0.326-6.300)	0.634
		AA	5(3.8)	4(3.7)	1.124(0.251-5.038)	0.878				
	rs2589144	GG	95(73.1)	58(53.7)						
	A/G	AG	30(23.1)	41(38.0)	0.541(0.293-0.998)	**0.049**	0.523(0.293-0.932)	**0.028**	0.543(0.168-1.757)	0.308
		AA	5(3.8)	9(8.3)	0.442(0.134-1.452)	0.178				

SUPPLEMENTARY TABLE 3: Genetic model associations between PTB and LTBI for selected SNPs.

Gene	SNP (m/M)	Genotypes	PTB patients	LTBI	Codominant (MM/Mm/mm)		Dominant (MM/Mm + mm)		Recessive (MM + Mm/mm)	
			N (%)		OR (95% CI)	*P**	OR (95% CI)	*P**	OR (95% CI)	*P**
	rs2672897	AA	59(45.4)	32(29.6)						
	G/A	GA	57(43.8)	57(52.8)	0.562(0.306-1.032)	0.063	0.539(0.302-0.963)	**0.037**	0.646(0.289-1.442)	0.286
		GG	14(10.8)	19(17.6)	0.462(0.191-1.114)	0.086				
	rs7209040	GG	62(47.7)	59(54.6)						
	A/G	AG	58(44.6)	40(37.0)	1.798(0.991-3.264)	0.054	1.694(0.961-2.984)	0.068	0.962(0.352-2.626)	0.939
		AA	10(7.7)	9(8.4)	1.259(0.442-3.584)	0.666				
	rs7224758	GG	89(68.5)	80(74.1)						
	A/G	AG	37(28.4)	25(23.1)	1.190(0.631-2.244)	0.592	1.186(0.645-2.180)	0.583	1.103(0.224-5.424)	0.904
		AA	4(3.1)	3(2.8)	1.155(0.233-5.728)	0.860				
	rs7503807	AA	69(53.0)	50(46.3)						
	C/A	CA	53(40.8)	48(44.4)	0.797(0.447-1.421)	0.442	0.768(0.441-1.338)	0.351	0.687(0.242-1.954)	0.482
		CC	8(6.2)	10(9.3)	0.617(0.209-1.817)	0.381				
TSC2	rs2074969	GG	100(76.9)	75(69.4)						
	A/G	AG	26(20.0)	30(27.8)	0.499(0.260-0.958)	**0.037**	0.512(0.273-0.960)	**0.037**	0.796(0.155-4.084)	0.785
		AA	4(3.1)	3(2.8)	0.648(0.124-3.395)	0.608				

PTB: pulmonary tuberculosis. LTBI: latent tuberculosis infection. 95% CI:95% confidence interval. OR: odds ratio. Rf: Reference. M: Major allele. m: minor allele. MM: homozygote of major allele. Mm: heterozygote. mm: homozygote of minor allele.

* Adjusted by sex and age, ＜0.05 is in bold.
