# Supplementary material for: Association of PI3K/AKT/mTOR pathway autophagy-related gene polymorphisms with pulmonary tuberculosis susceptibility in a Chinese population
Source: Rev Soc Bras Med Trop. 2023 Jul 24;56:e0104-2023. doi: 10.1590/0037-8682-0104-2023 (PMC10367219; doi:10.1590/0037-8682-0104-2023)
Supplement: Supplementary file 5 [file 1678-9849-rsbmt-56-e0104-2023-supp5.pdf]



SUPPLEMENTARY TABLE 4: Genetic model associations between LTBI and HC for selected SNPs.

Gene	SNP (m/M)	Genotypes	LTBI	HC	Codominant (MM/Mm/mm)		Dominant (MM/Mm + mm)		Recessive (MM + Mm/mm)	
			N (%)		OR (95% CI)	P^*	OR (95% CI)	P^*	OR (95% CI)	P^*
AKT1	rs1130233	AA	34(31.5)	55(33.7)	Rf		Rf		Rf	
	G/A	GA	58(53.7)	84(51.5)	1.106(0.635-1.929)	0.721	1.070(0.628-1.824)	0.803	0.889(0.434-1.819)	0.747
		GG	16(14.8)	24(14.8)	0.946(0.427-2.095)	0.892				
	rs11848899	CC	78(72.2)	97(60.3)						
	A/C	AC	28(25.9)	58(36.0)	0.558(0.318-0.979)	**0.042**	0.539(0.312-0.931)	**0.027**	0.429(0.080-2.286)	0.321
		AA	2(1.9)	6(3.7)	0.355(0.066-1.919)	0.229				
	rs12432802	AA	29(26.8)	40(24.6)						
	G/A	GA	53(49.1)	91(55.8)	0.828(0.455-1.507)	0.537	0.873(0.495-1.541)	0.640	1.128(0.612-2.081)	0.699
		GG	26(24.1)	32(19.6)	0.995(0.480-2.064)	0.990				
	rs2494738	AA	32(29.6)	44(27.0)						
	G/A	GA	53(49.1)	88(54.0)	0.745(0.414-1.338)	0.324	0.777(0.446-1.356)	0.777	1.056(0.564-1.977)	0.864
		GG	23(21.3)	31(19.0)	0.872(0.419-1.814)	0.713				
	rs2494743	GG	59(54.6)	81(49.7)						
	A/G	AG	38(35.2)	71(43.6)	0.670(0.391-1.148)	0.145	0.764(0.460-1.268)	0.297	1.677(0.680-4.134)	0.262
		AA	11(10.2)	11(6.7)	1.409(0.555-3.580)	0.471				
AKT2	rs1991823	AA	41(38.0)	55(33.7)						
	G/A	GA	54(50.0)	89(54.6)	0.875(0.508-1.506)	0.630	0.901(0.534-1.519)	0.695	1.112(0.513-2.410)	0.788
		GG	13(12.0)	19(11.7)	1.026(0.444-2.373)	0.951				
	rs4803320	GG	46(43.0)	66(40.5)						
	A/G	AG	50(46.7)	83(50.9)	0.929(0.547-1.578)	0.784	0.967(0.581-1.609)	0.897	1.241(0.528-2.920)	0.621
		AA	11(10.3)	14(8.6)	1.192(0.484-2.938)	0.702				
mTOR	rs12122605	GG	62(57.4)	97(59.5)						
	A/G	AG	39(36.1)	58(35.6)	1.105(0.650-1.879)	0.713	1.146(0.690-1.903)	0.600	1.383(0.476-4.020)	0.551
		AA	7(6.5)	8(4.9)	1.437(0.485-4.260)	0.513				
	rs2536	AA	84(77.8)	135(82.8)						

SUPPLEMENTARY TABLE 4: Genetic model associations between LTBI and HC for selected SNPs.

Gene	SNP (m/M)	Genotypes	LTBI	HC	Codominant (MM/Mm/mm)		Dominant (MM/Mm + mm)		Recessive (MM + Mm/mm)	
			N (%)		OR (95% CI)	P^*	OR (95% CI)	P^*	OR (95% CI)	P^*
	G/A	GA	23(21.3)	26(16.0)	1.577(0.831-2.994)	0.163	1.525(0.815-2.854)	0.187	0.788(0.067-9.239)	0.850
		GG	1(0.9)	2(1.2)	0.861(0.073-10.148)	0.905				
	rs3806317	AA	84(78.5)	120(74.5)						
	G/A	GA	19(17.8)	39(24.2)	0.649(0.344-1.226)	0.183	0.768(0.422-1.398)	0.388	3.659(0.642-20.860)	0.144
		GG	4(3.7)	2(1.2)	3.346(0.584-19.175)	0.175				
PIK3CA	rs1607237	GG	60(55.6)	82(50.3)						
	A/G	AG	40(37.0)	69(42.3)	0.783(0.462-1.325)	0.362	0.809(0.490-1.336)	0.408	1.078(0.409-2.841)	0.879
		AA	8(7.4)	12(7.4)	0.972(0.360-2.625)	0.955				
	rs2677760	GG	66(61.1)	93(57.0)						
	A/G	AG	37(34.3)	65(39.9)	0.854(0.503-1.449)	0.559	0.891(0.534-1.488)	0.660	1.439(0.391-5.302)	0.584
		AA	5(4.6)	5(3.1)	1.352(0.361-5.060)	0.654				
PTEN	rs2299939	CC	73(67.6)	105(64.4)						
	A/C	AC	32(29.6)	52(31.9)	1.055(0.606-1.836)	0.849	1.026(0.601-1.754)	0.924	0.780(0.185-3.280)	0.735
		AA	3(2.8)	6(3.7)	0.794(0.186-3.378)	0.755				
	rs741804	AA	74(68.5)	117(72.2)						
	C/A	CA	30(27.8)	42(25.9)	1.270(0.717-2.248)	0.412	1.328(0.766-2.304)	0.313	1.932(0.408-9.144)	0.406
		CC	4(3.7)	3(1.9)	2.060(0.431-9.834)	0.365				
RHEB	rs2299962	GG	51(47.2)	76(46.6)						
	A/G	AG	46(42.6)	75(46.0)	0.886(0.524-1.500)	0.653	0.956(0.579-1.578)	0.859	1.472(0.616-3.513)	0.384
		AA	11(10.2)	12(7.4)	1.388(0.560-3.436)	0.479				
	rs3789817	GG	40(37.4)	43(26.4)						
	A/G	AG	47(43.9)	82(50.3)	0.584(0.327-1.045)	0.070	0.567(0.330-0.975)	**0.040**	0.730(0.390-1.364)	0.323
		AA	20(18.7)	38(23.3)	0.531(0.260-1.083)	0.082				
	rs6972955	AA	26(24.3)	50(30.7)						
	C/A	CA	58(54.2)	88(54.0)	1.264(0.697-2.292)	0.441	1.394(0.789-2.464)	0.253	1.597(0.840-3.035)	0.154

SUPPLEMENTARY TABLE 4: Genetic model associations between LTBI and HC for selected SNPs.

Gene	SNP (m/M)	Genotypes	LTBI	HC	Codominant (MM/Mm/mm)		Dominant (MM/Mm + mm)		Recessive (MM + Mm/mm)	
			N (%)		OR (95% CI)	P^*	OR (95% CI)	P^*	OR (95% CI)	P^*
		CC	23(21.5)	25(15.3)	1.866(0.875-3.982)	0.107				
RPS6KB1	rs180515	AA	34(31.5)	46(28.4)						
	G/A	GA	50(46.3)	83(51.2)	0.813(0.456-1.452)	0.485	0.856(0.497-1.476)	0.576	1.095(0.595-2.016)	0.771
		GG	24(22.2)	33(20.4)	0.964(0.475-1.954)	0.919				
	rs180519	AA	32(29.6)	60(36.8)						
	G/A	GA	55(50.9)	71(43.6)	1.562(0.880-2.770)	0.128	1.489(0.869-2.551)	0.148	1.020(0.543-1.917)	0.950
		GG	21(19.5)	32(19.6)	1.326(0.646-2.724)	0.442				
RPTOR	rs10871489	AA	61(56.5)	100(61.3)						
	G/A	GA	41(38.0)	53(32.5)	1.303(0.766-2.216)	0.328	1.267(0.763-2.105)	0.361	0.966(0.331-2.821)	0.949
		GG	6(5.5)	10(6.2)	1.067(0.358-3.180)	0.907				
	rs11651587	AA	39(36.1)	64(39.3)						
	G/A	GA	56(51.9)	74(45.4)	1.305(0.755-2.254)	0.340	1.165(0.693-1.958)	0.564	0.677(0.321-1.427)	0.305
		GG	13(12.0)	25(15.3)	0.785(0.350-1.759)	0.556				
	rs11654508	AA	36(33.3)	69(42.4)						
	G/A	GA	58(53.7)	70(42.9)	1.717(0.989-2.982)	0.055	1.622(0.958-2.744)	0.072	0.975(0.473-2.011)	0.945
		GG	14(13.0)	24(14.7)	1.326(0.600-2.935)	0.486				
	rs12602885	GG	56(51.8)	76(46.6)						
	A/G	AG	46(42.6)	77(47.2)	0.862(0.512-1.453)	0.577	0.868(0.523-1.441)	0.584	0.985(0.340-2.851)	0.978
		AA	6(5.6)	10(6.2)	0.914(0.306-2.730)	0.872				
	rs2090204	CC	65(60.2)	102(62.6)						
	A/C	AC	39(36.1)	52(31.9)	1.226(0.719-2.091)	0.455	1.150(0.688-1.924)	0.594	0.656(0.190-2.261)	0.504
		AA	4(3.7)	9(5.5)	0.707(0.202-2.478)	0.588				
	rs2589144	GG	58(53.7)	94(57.7)						
	A/G	AG	41(38.0)	58(35.6)	1.038(0.608-1.774)	0.891	1.101(0.665-1.824)	0.709	1.426(0.559-3.639)	0.457
		AA	9(8.3)	11(6.7)	1.447(0.555-3.772)	0.450				

SUPPLEMENTARY TABLE 4: Genetic model associations between LTBI and HC for selected SNPs.

Gene	SNP (m/M)	Genotypes	LTBI	HC	Codominant (MM/Mm/mm)		Dominant (MM/Mm + mm)		Recessive (MM + Mm/mm)	
			N (%)		OR (95% CI)	*P**	OR (95% CI)	*P**	OR (95% CI)	*P**
	rs2672897	AA	32(29.6)	55(33.8)						
	G/A	GA	57(52.8)	70(42.9)	1.363(0.768-2.419)	0.290	1.201(0.700-2.060)	0.507	0.739(0.394-1.389)	0.348
		GG	19(17.6)	38(23.3)	0.890(0.433-1.830)	0.752				
	rs7209040	GG	59(54.6)	72(44.2)						
	A/G	AG	40(37.0)	74(45.4)	0.651(0.383-1.107)	0.113	0.631(0.381-1.046)	0.074	0.668(0.274-1.626)	0.374
		AA	9(8.4)	17(10.4)	0.547(0.217-1.377)	0.200				
	rs7224758	GG	80(74.1)	125(76.7)						
	A/G	AG	25(23.1)	36(22.1)	1.132(0.622-2.061)	0.686	1.214(0.680-2.168)	0.511	2.750(0.432-17.485)	0.284
		AA	3(2.8)	2(1.2)	2.831(0.443-18.107)	0.272				
	rs7503807	AA	50(46.3)	69(42.3)						
	C/A	CA	48(44.4)	74(45.4)	0.937(0.549-1.597)	0.810	0.902(0.541-1.501)	0.690	0.789(0.347-1.794)	0.573
		CC	10(9.3)	20(12.3)	0.763(0.320-1.817)	0.541				
TSC2	rs2074969	GG	75(69.4)	100(61.3)						
	A/G	AG	30(27.8)	53(32.5)	0.899(0.515-1.569)	0.707	0.835(0.489-1.428)	0.511	0.504(0.132-1.932)	0.318
		AA	3(2.8)	10(6.2)	0.486(0.125-1.887)	0.297				

LTBI: latent tuberculosis infection. HC: health control. 95% CI:95% confidence interval. OR: odds ratio. Rf: Reference. M: Major allele. m: minor allele. MM: homozygote of major allele. Mm: heterozygote. mm: homozygote of minor allele.

* Adjusted by sex and age, ＜0.05 is in bold.
