# Supplementary material for: Association of PI3K/AKT/mTOR pathway autophagy-related gene polymorphisms with pulmonary tuberculosis susceptibility in a Chinese population
Source: Rev Soc Bras Med Trop. 2023 Jul 24;56:e0104-2023. doi: 10.1590/0037-8682-0104-2023 (PMC10367219; doi:10.1590/0037-8682-0104-2023)
Supplement: Supplementary file 6 [file 1678-9849-rsbmt-56-e0104-2023-supp6.pdf]

SUPPLEMENTARY TABLE 5: The SNP-SNP interaction models identified by the GMDR method.

Interaction model	Test accuracy (%)	*P*
rs11848899	50.45	**0.0107**
rs2090204, rs2589144	47.72	0.3770
rs3789817, rs12602885, rs2672897	48.26	0.1719
rs12432802, rs3789817, rs2672897, rs11654508	48.36	0.1719
rs12432802, rs3789817, rs7503807, rs2672897, rs11654508	47.55	0.0547
